# Does emotional valence affect cognitive performance and neurophysiological response during decision making? A preliminary study

**DOI:** 10.3389/fnins.2024.1408526

**Published:** 2024-08-09

**Authors:** Michela Balconi, Katia Rovelli

**Affiliations:** ^1^International research center for Cognitive Applied Neuroscience, Università Cattolica del Sacro Cuore, Milan, Italy; ^2^Research Unit in Affective and Social Neuroscience, Department of Psychology, Università Cattolica del Sacro Cuore, Milan, Italy

**Keywords:** emotional valence, decision making, EEG, social stress test, cognitive performance

## Abstract

This study investigated the impact of the emotional valence of external situations (EVES) on cognitive performance and electrophysiological (EEG) responses during decision-making. 26 healthy adults underwent a modified version of the Trier social stress test, performing five interview-style discourses. Each discourse entailed preparing a speech under increasingly stressful conditions. Participants were also exposed to gradually increasing EVES (i.e., an examining committee displaying progressively more negative-connoted emotional facial expressions). In addition, after each speech, participants completed an arithmetic task to test how emotional manipulation affected cognitive performance. Behavioral data (preparation times) and EEG data (frequency bands) were collected to assess stress regulation, stress resilience, and cognitive performance. The results indicate that EVES significantly influenced stress regulation and resilience, as reflected in the behavioral data. Neurophysiological findings showed increased parietal lobe activity (P4) in the theta and delta bands with rising emotional valence, plateauing from the preparation of the second discourse onward. This suggests enhanced emotional processing and attentional demands. However, gamma band activity decreased in P4 during the preparations for the two discourses following the first, indicating a shift of cognitive resources from higher cognitive functions to emotional processing. This highlights the cognitive cost of maintaining performance and stress regulation under emotionally charged conditions. Such findings suggest that emotional valence modulates cognitive performance and that specific neural mechanisms are involved in managing stress responses. The findings underscore the complex relationship between emotion, cognition, and neural mechanisms, offering valuable insights for stress regulation and resilience, and enhancing performance under pressure.

## 1 Introduction

In everyday life, individuals are constantly exposed to several external stimuli and impactful situations, some of which are of particular significance due to their emotional valence. Emotional valence is a critical factor when examining cognitive functioning (Ferri et al., 2010; Kauschke et al., 2019). Recent research has revealed how exposure to negative audiovisual stimuli impacts information processing times and performance, compared to neutral stimuli (Bowling, 2015; Román et al., 2015). Researchers argue that this effect is driven by the allocation of attentional resources demanded by these stimuli (Plancher et al., 2019), in accordance with the selective visual attention research model proposed by Treisman and Gelade (1980). Aligning with prior research and theories on emotional biases (Pool et al., 2016), the tendency of people to respond more positively than negatively to mild emotional stimuli (i.e., positivity offset) and the tendency to respond more strongly to very negative stimuli than to matched positive stimuli (i.e., negativity bias) can be attributed to emotional and attentional biases (Vanutelli et al., 2017; Balconi and Pozzoli, 2005; Yuan et al., 2019). These biases can significantly influence our perception, attention, memory (Norris et al., 2011), our emotional regulation, cognitive control load, our actions and executive functions (Goldstein and Brockmole, 2002). In particular, the relationship between emotions and executive functions has been extensively studied, considering the common neural circuits involved in both processes, including the ventromedial prefrontal cortex and the amygdala (Tranel, 2002; Manuel et al., 2020).

However, there has been little research focused on understanding how the EVES impact decision making and cognitive performance. Additionally, to the best of our knowledge, no study has investigated individuals’ capacity to plan a performance when confronted with progressively intensifying EVES.

To examine these aspects, we used a modified version of the Trier social stress test (TSST; Allen et al., 2017), named social stress test (SST).

The SST used in this research required participants to engage in the preparation and delivery of five different discourses (D_1–5_). For each discourse, participants were asked to read a request, specific to that discourse (see Table 1), and then prepare a speech that aligned with the request. This phase (Preparation phase, Pp) was chosen as the most critical part of the task, as participants were informed beforehand that achieving a higher score required them to prepare their speech as quickly as possible.

**TABLE 1:** Description of the requests for each discourse (REQ_1–5_).

REQs	Text
REQ_1_	We ask you to prepare the best presentation of yourself
REQ_2_	We kindly request you to prepare a situation in which you encountered difficulties in making a decision during your academic/professional career or during previous internships
REQ_3_	We kindly request you to provide an instance from your academic/professional environment where you found yourself in a challenging situation without any support in making a decision
REQ_4_	We kindly request you to reflect on a situation in which you found yourself making a decision within a university context or during previous work experiences, assuming full responsibility for it, including on behalf of others, without the opportunity to consult them
REQ_5_	We ask you to describe a situation in which you found yourself taking a critical decision alone in the academic/professional context, by taking the full responsibility of it and in complete disagreement with the rest of the group

Instead, to examine how healthy adults respond to different EVES, participants were asked to watch a video depicting an evaluation committee immediately after each preparation. Unlike the TSST, which exclusively features negative emotional valence, in this modified version, the intensity and the valence of the EVES was gradually manipulated through the examining committee’s facial expressions across five distinct phases (see Supplementary File 1).

Subsequently, participants were asked to orally present their speech.

The individuals’ capacity to plan a performance with progressively intensifying EVES, was assessed by recording and examining the length of the Pp, i.e., the Preparation time (P_T_). This was defined as the interval between the key press that signals the start of the Pp and the subsequent key press that marks the end of this phase. The P_T_ had a maximum duration of 120 s.

We computed two indexes: the stress regulation index (Reg_Stress_)—defined as the ability to effectively manage physiological and psychological stress reactions, playing a central role in modulating neurocognitive efficiency (Williams et al., 2009; Crum et al., 2020)—and the stress resilience index (Res_Stress_), defined as the ability to respond promptly and appropriately to more acute and intense stress conditions (Fleshner et al., 2011; Khayrutdinov et al., 2020).

Furthermore, we also examined the impact of the increasing EVES measuring the outcome of the participants’ cognitive performance. Following each speech, they were asked to complete an arithmetic task (aT), and performance accuracy was recorded.

Additionally, to gain a better understanding of the impact of valence on neurocognitive performance, we recorded brain activity via electroencephalography (EEG frequency band) (Balconi et al., 2015b). Different frequency bands have significant functional roles in modulating attention, cognitive workload, emotional engagement, and higher-order cognitive functions. Thus, by exploring changes of bands we aim to get insights into the neural mechanisms handle the EVES. For instance, gamma oscillations are essential for integrating sensory information and emotional processing, while alpha and beta bands are involved in attention modulation and cognitive workload, respectively (Vernet et al., 2012; Tu et al., 2015). The EEG analyses aimed to detect differences in frequency bands across task phases according to different emotional and cognitive contexts.

According to the above-cited literature, we hypothesize that exposure to different experimental contexts would induce an attentional bias toward heightened emotional valence (i.e., negativity bias); (Kauschke et al., 2019) leading to decreased P_T_. The reduction in P_T_ may be attributed to negative EVES, which enhance cognitive processes such as attention and perception, thereby eliciting faster responses. We also hypothesized that performance in the aT would improve following a stressful situation, due to the higher allocation of attentional resources that foster regulation and resilience.

## 2 Materials and methods

### 2.1 Sample

To determine the minimum required sample size, *a priori* power analysis for repeated measures ANOVA was conducted, revealing that a total sample size of 21 (alpha error probability = 0.05; power = 0.80; number of groups = 1; Effect size *f* = 0.25), was necessary to detect a significant within-effect or interaction between factors (G*Power 3.1; Faul et al., 2007). Thus, a total of 26 healthy adults were selected as participants for this study (M_age_ = 23.038, SD_age_ = 1.455, age range: 22–28, N_female_ = 16). All participants were Caucasian residents of Lombardy, Italy, with a mean educational attainment of 16.38 years (SD = 1.04). They were naïve to the purposes of the experiment, right handed, and had a normal or corrected visual and auditory acuity. Furthermore, they have no significant distress or burnout, no history of neurological or psychiatric illnesses, no current psychoactive substance therapy, and no major stressful life events in the past six months.

All participants provided written informed consent without compensation. The study was approved by the Ethics Committee of the Department of Psychology (approval code: 2021 PhDTD), Catholic University of The Sacred Heart, Milan, Italy, and was conducted in adherence to the guidelines outlined in the Declaration of Helsinki (2013) and according to the General Data Protection Regulation–Reg. UE 2016/679 and its ethical guidelines.

### 2.2 Experiment procedure

The experiments took place in a moderately lit room. Participants were seated facing a workstation equipped with a computer and a mouse, positioned 100 cm away. They were informed they would participate in a digital job interview to assess their ability to respond to occupational positions in the future, competing with other candidates. They were also informed that their performance would be evaluated by a virtual committee, i.e., participants were aware that these was a fictitious interview. After these instructions, they were asked to sign the informed consent.

Before starting the experiment, the EEG baseline activity was recorded with eyes closed and open. Autonomic activity was also monitored during the experiment. Additional information is reported in the Supplementary File 1.

A web-based experiment management platform (Qualtrics XM platform; Qualtrics LLC, Provo, UT, USA) was employed for administration. For each of the five discourses, participants were asked to: (i) read the requests (REQ_1–5_, Table 1); (ii) prepare the discourses (Pp_1–5_; maximum time: 120 s); (iii) watch the evaluation committee video (see Supplementary File 1); and (iv) deliver the speech (maximum time: 60 s). Finally, after each speech, participants engaged in the aT (aT_1–5_) of similar difficulty to evaluate the effect of emotional manipulation on cognitive performance. These tasks required participants to verbally subtract numbers in a series, aiming to generate as many sequences as possible within a 30-s timeframe (e.g., aT_1_: “*Begin with the number 200 and subtract the number 6 consecutively*”).

**FIGURE 1:**
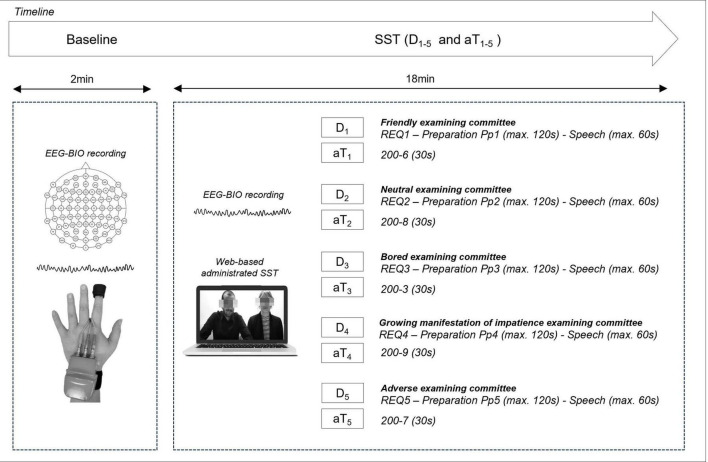
Sequence of the discourses (D_1–5_) and arithmetic Task (aT_1–5_) within the experimental procedure. For every discourse, the participants were required: (i) to read the requests (REQ_1–5_); (ii) to prepare the discourses (Pp_1–5_; max-time: 120 s); (iii) to watch the evaluation committee videos; (iv) to present the discourses (max-time: 60 s). EEG and autonomic activity were monitored from the baseline throughout the SST together with behavioral data recording.

The experimental procedure lasted approximately 50 min (Figure 1, see also Supplementary File 1).

Three experts in the psychology of emotions and stress who did not take part in the experiment assessed the emotional situations of the study to ensure their consistency and reliability in eliciting the intended experimental outcomes.

### 2.3 Behavioral data acquisition and processing

To assess the influence of EVES on neurocognitive response two sets of behavioral data were collected and analyzed.

First, P_T_ of each discourse were computed. These data were then processed offline to derive Reg_Stress_ and Res_Stress_ scores. We used objective and qualitative criteria to evaluate the relevance, coherence of content, and appropriateness of each discourse. Discourses failing to meet productivity criteria—characterized by lack of substance, inclusion of off-task or inconsistent topics—were excluded from analysis and deleted from the database (0.77% data exclusion rate, see Supplementary File 1). This procedure ensured content equivalence across the five analyzed discourses, enabling an objectively comparable assessment of performance originating from the Pp.

To compute Reg_Stress_ and Res_Stress_ scores, we followed three steps. First, each raw score (see Supplementary Table 1a: P_Traw1_; P_Traw2_; P_Traw3_; P_Traw4_; P_Traw5_) was assigned a score on a scale from 1 (low performance) to 5 (high performance): P_T1_, P_T2_, P_T3_, P_T4_, P_T5_. The scoring was determined through an analysis of the normal distribution of results from a preliminary test (*N* = 131), conducted before the current research.

Subsequently, data were converted into two distinct scores:

(a) a score representing stress regulation, calculated as follows:


$${\text{Reg}}_{\text{Stress}}\,\mathrm{score}=\frac{\text{P}\,\mathrm{T1}+\text{P}\,\mathrm{T2}+\text{P}\,\mathrm{T3}+\text{P}\,\mathrm{T4}+\text{P}\,\mathrm{T5}}{5}$$

(b) a score representing stress resilience, calculated as follow:


$${\text{Res}}_{\text{Stress}}\,\mathrm{score}=\left|\left(\text{P}\,\mathrm{T4}-\text{P}\,\mathrm{T5}\right)\right|-{\mathrm{Reg}}_{\text{Stress}}\,\mathrm{score}$$

Finally, to further enhance the compatibility and comparability of our findings with future studies, we converted the Reg_Stress_ and Res_Stress_ scores into deciles. This approach allows for a more standardized and reliable measure, facilitating the interpretation and comparison of results across studies (see also Supplementary File 1).

Secondly, for each aT, an accuracy index was computed by comparing the number of correct sequences with the total number of sequences performed by each participant in the series.

### 2.4 Electrophysiological data acquisition and processing

Before starting the experiment, we performed a resting-state Recording the EEG with the eyes open and closed. Each condition lasted 120 s (Christie et al., 2017; Ceh et al., 2020; Angioletti and Balconi, 2022).

EEG data for baseline and for the Pp_1–5_ of the SST was collected using an 18-channel DC amplifier (SYNAMPS) and NEUROSCAN 4.2 software. ElectroCap with Ag/AgCl electrodes was used, placed on 18 scalp sites following the 10/20 system, referencing the earlobes (Jasper, 1958). Furthermore, two electrooculographic electrodes were placed above and below the left eye of each participant, avoiding visual interference. Data were sampled at 1,000 Hz and filtered with a 50 Hz notch input filter. Electrode impedance was checked before data collection, ensuring it remained below 5 kΩ. Following, data from eyes-open and eyes-closed resting states and related to the Pp_1–5_ were processed offline (IIR bandpass filter 0.1–50 Hz, 48 dB/octave) using Vision Analyzer2 software (Brain Products GmbH, Gilching, Germany), and were corrected with an ICA-based algorithm (Jung et al., 2000). Following EOG correction and meticulous visual inspection, only segments devoid of muscle or eye artifacts and other disturbances were considered (rejected epochs, 3%; average number of epochs selected for Pp: Pp1—20.8; Pp2—13.7; Pp3—15.6; Pp4—19.1; Pp5—13.1). All the electrodes were used for the successive statistical analysis (AFz, Fp1, F7, F3, Fz, F4, F8, Fp2, T7, C3, Cz, C4, T8, P3, Pz, P4, O1, O2). Successively, the data were epoched with a 2,000 ms window to maintain EEG data integrity. Artifact-free data were then used to calculate condition-specific Power Spectral Density (PSD) using fast Fourier transform (Hamming window, resolution: 0.5 Hz). Lastly, average PSD for the main EEG frequency bands [delta (0.5–3.5 Hz); theta (4–7.5 Hz); alpha (8–12.5 Hz); beta (13–30 Hz); and gamma (30.5–50 Hz)] was extracted for each phase considered. Additionally, all task-related data were normalized to the eyes-open baseline of each participant.

### 2.5 Statistical analysis

Repeated measures ANOVAs (analysis of variance) were applied to behavioral and EEG data.

For behavioral data, a repeated measures ANOVA with Pp (5 levels) as the within-independent subject factor was applied to the P_T_ scores, Reg_Stress_ and Res_Stress_, as dependent variables. For the aT, a repeated measures ANOVA with Task (5 levels) as the within-subject factor was applied to the accuracy index. Finally, correlation analyses (Pearson correlation coefficients) between Reg_Stress_ and Res_Stress_, respectively, and the accuracy index of the five aT were computed.

For EEG data, five repeated measures ANOVAs considering Electrode (18) and Pp (5) as within-subject independent variables were carried out for the five different frequency bands.

Degrees of freedom in all ANOVA tests were adjusted using the Greenhouse–Geisser epsilon when appropriate. Significant interactions were explored using pairwise comparisons with Bonferroni correction. Partial eta squared values were computed to estimate the effect sizes. IBM SPSS 29 (IBM Corp., Chicago, IL, USA) was used for the statistical analysis.

The normal distribution of the data was assessed by using skewness and kurtosis test. Furthermore, as preliminary analysis showed no statistically significant main effects or interactions concerning gender; this variable was excluded from subsequent analyses.

## 3 Results

All the descriptive statistics are reported in the Supplementary Table 1.

### 3.1 Behavioral and correlational results

**FIGURE 2:**
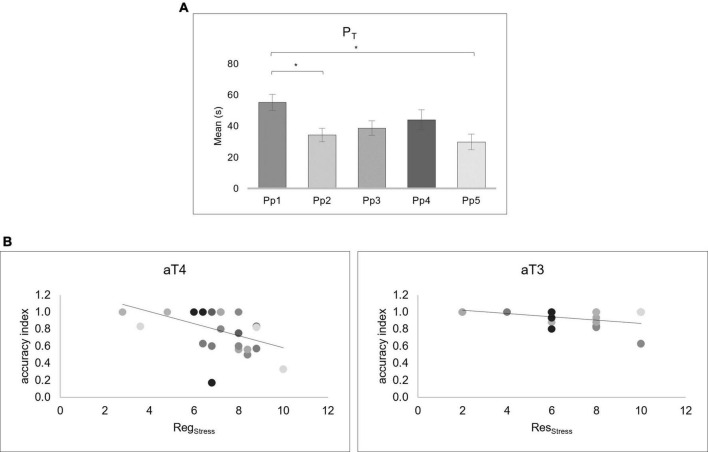
Behavioral Preparation time (P_T_) scores results and correlations between the accuracy index of the arithmetic Task (aT) and Reg_Stress_ and Reg_Stress_ indexes. **(A)** The bar graph shows the P_T_ scores for each preparation phase (Pp). Bars represent ± 1 Standard Error and stars (*) mark statistically significant comparisons. **(B)** The scatter plots at the left display the correlation between the accuracy index and the Reg_Stress_ score in the aT4. The scatter plots at the right display the correlation between accuracy index and Res_Stress_ score in the aT3. The different colors of the dots correspond to each Pp.

The analysis on the behavioral data showed significant main effect of P_T_ [*F*_(2.2_,_ 55.5)_ = 5.455, *p* = 0.005, *ηp*^2^ = 0.179]. *Post hoc* tests showed that P_T1_ was longer than P_T2_ (*p* = 0.035; 95% CI [0.94, 40.88]), and P_T5_ (*p* = 0.018; 95% CI [3.07, 47.77]) (Figure 2A).

No significant differences were found for the ANOVAs performed on the accuracy index of the aT.

The Pearson correlation analyses revealed a negative correlation between the Reg_Stress_ and accuracy index in aT_4_ (*r* = −0.465, *p* = 0.017) and a negative correlation between the Res_Stress_ and accuracy index in aT_3_ (*r* = −0.453, *p* = 0.020) (Figure 2B).

### 3.2 Electroencephalographic results

The repeated measures ANOVAs performed on each of the five different frequency bands (delta, theta, alpha, beta, and gamma) are reported.

**FIGURE 3:**
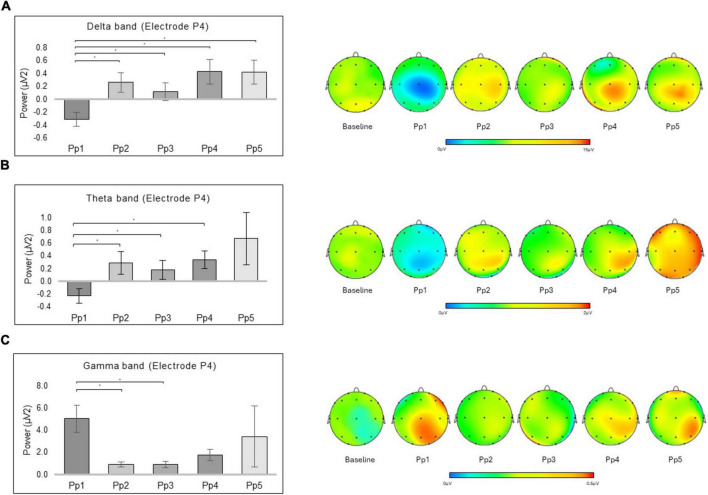
EEG results. The bar graph shows the significant differences for the delta, theta, and gamma bands in Electrode × Preparation. For all graph, bars represent ± 1 Standard Error and stars (*) mark statistically significant comparisons. For topographic EEG maps, red represented an increase in power for the considered frequency band. **(A)** The bar graph displays the significantly higher mean values in P4 for the Pp_2_, Pp_3_, Pp_4_, and Pp_5_, compared to the Pp_1_ for the delta band. The topographic EEG maps of spectral power density for the delta band are reported (Software: Vision Analyzer2, Brain Products GmbH, Gilching, Germany). **(B)** The bar graph displays the significantly higher mean values in P4 for the Pp_2_, Pp_3_, and Pp_4_, compared to the Pp_1_ for theta band. The topographic EEG maps of spectral power density for the theta band are reported. **(C)** The bar graph displays the significantly higher mean values in P4 for the Pp_1_ compared to the Pp_2_ and the Pp_3_ for the gamma band. The topographic EEG maps of spectral power density for the gamma band are reported.

For delta band, a significant interaction effect was revealed for Electrode × Preparation [*F*_(68_,_ 1292)_ = 2.158, *p* ≤ 0.001, *ηp*^2^ = 0.102] (Figure 3A). Significant pairwise comparisons revealed higher mean values in P4 for the Pp_2_ (*p* = 0.001; 95% CI [−0.93, −0.21]), Pp_3_ (*p* = 0.012; 95% CI [−0.78, −0.06]), Pp_4_ (*p* = 0.003; 95% CI [−1.27, −0.20]), Pp_5_ (*p* = 0.006; 95% CI [−1.30, −0.16]) compared to the Pp_1._

In the same way, for theta band, a significant interaction effect was revealed for Electrode × Preparation [*F*_(68_,_ 1292)_ = 1.940, *p* ≤ 0.001, *ηp*^2^ = 0.093] (Figure 3B). Significant pairwise comparisons revealed higher mean values in P4 for the Pp_2_ (*p* = 0.003; 95% CI [−0.90, −0.15]), Pp_3_ (*p* = 0.017; 95% CI [−0.77, −0.05]), Pp_4_ (*p* = 0.001; 95% CI [−0.94, −0.20]), compared to the Pp_1_.

Lastly, for gamma band, a significant interaction effect was revealed for Electrode × Preparation [*F*_(68_,_ 1292)_ = 1.508, *p* = 0.006, *ηp*^2^ = 0.074] (Figure 3C). Significant pairwise comparisons revealed higher mean values in P4 for Pp_1_ compared to the Pp_2_ (*p* = 0.025; 95% CI [0.35, 7.91]), and Pp_3_ (*p* = 0.031; 95% CI [0.27, 7.80]).

No other significant differences were found for the ANOVAs performed on the EEG Pp data, as well as on the alpha and beta bands.

All the results for P4 are reported in the Supplementary Table 2. Plot of PSD across frequency at P4 for each of the five discourses are reported in the Supplementary Figure 2, in the Supplementary File 1.

## Discussion

This pilot study explored the relationship between the progressively increasing negativity valence during the presentation of different ecologically EVES and the corresponding behavioral and neurophysiological responses.

Behavioral findings revealed a decrease in the P_T_, according to heightened negativity. This is more evident in Pp_2_, when the emotional valence starts to become more negative and in Pp_5_, when the negative valence is maximal. These findings can be explained by an increase of attentional and emotional biases in participants when the context becomes more and more negative. Such interpretation aligns with previous research indicating that low-negative valence situations tend to elicit a positive response, whereas high-negative emotional valence situations often result in a negativity bias. This is primarily due to the heightened attention and reactivity typically evoked by negative situations, leading to shorter P_T_ (Vanutelli et al., 2017; Balconi and Pozzoli, 2005; Yuan et al., 2019).

The behavioral P_T_ data show a *plateau* effect starting from the Pp_2_. This means there was no significant variation in performance after the first discourse, although the negative intensity progressively increased. This phenomenon might be related to the “negativity saturation threshold.” Although the degree of negativity increases, participants’ subjective perception does not seem to increase further. This suggests that there is a limit beyond which negative emotional situations stabilize. This is supported by the fact that significant differences were only observed between Pp_1_ and Pp_2_, and between Pp1 and Pp5, but not between the preparations after the first (for example, no difference was found between Pp_5_ and Pp_4_, or between Pp_4_ and Pp_3_). These findings imply that, after reaching a certain point, additional negative stimuli do not elicit a proportionate increase in the participants’ subjective negative experience, thereby supporting the concept of a negativity saturation threshold.

Instead, the Reg_Stress_ and Res_Stress_ indexes did not show significant differences. Considering the functional significance attributed to such indexes, this suggests that people can respond similarly despite the emotional situations turn to be more negative. However, this stability comes at a cost. Some researchers suggest that the increasing negativity of emotional situations could potentially reduce people’s ability to effectively process information, lowering performance (Li et al., 2022). In fact, considering cognitive performance, we found a negative correlation between the Reg_Stress_ index and accuracy in the fourth arithmetic task, as well as between the Res_Stress_ index and accuracy in the third arithmetic task. This result could be due to the fact that people, to maintain consistent levels of stress regulation and resilience, allocate a significant amount of cognitive and emotional resources in this process, subtracting cognitive resources reducing the ability to effectively perform complex cognitive tasks.

In terms of neurophysiological findings, an augmentation in parietal lobe activity (P4), has been noted in the theta and delta bands as the negative valence of situations increases. This trend seems to plateau from Pp_2_ onward, paralleling the pattern observed in the P_T_ data. It has been shown that an increase in parietal lobe activity in theta band and delta band indicates emotional central processing, attentional demands, as well as stimulus evaluation (Balconi and Pozzoli, 2009; Balconi et al., 2015b; Balconi and Vanutelli, 2017; Balconi and Angioletti, 2021). Additionally, the parietal lobe is involved in top-down attentional processes guided by episodic retrieval goals, while ventral parts of the parietal lobe support bottom-up attentional processes captured by retrieval output (Dobbins et al., 2012). Instead, the inferior parietal lobe is involved in matching perceptual information about observed actions with motoric representations, enabling the understanding of actions and intentions (Thakkar et al., 2014). The heightened activity observed in this region indicate a greater level of engagement in the assessment and interpretation of EVES (Viviani, 2013). In fact, the processing of EVES involves heightened theta and delta activity, which aids in reinterpreting the stimulus and regulating emotional responses over time (Lapomarda et al., 2022). This regulation is primarily supported by visuospatial networks located in the right parieto-occipital lobe (Wang et al., 2010; Maksimenko et al., 2018). This explains the involvement of electrode P4, representing a region of the parietal lobe known to be involved in complex cognitive processes, including those related to the integration of information (Gonzalez and Flindall, 2015; García-Martínez et al., 2019; Steber et al., 2020).

Conversely, a decrease in gamma-band activity associated with higher cognitive demand, and metacognition (Herrmann et al., 2010), was observed in P4 in Pp_2_ and Pp_3_ compared to Pp_1_. The reduction in gamma activity during the central phases of the SST may reflect a redistribution of cognitive resources toward emotional processing at the expense of higher cognitive functions, as also supported by the negative correlations between accuracy in aT in central tasks and the two stress response indexes. This can represent a “cost to pay” for maintaining an adequate performance and stress regulation to the increasing EVES when the interaction becomes more and more engaging because of a greater negative charge.

These findings should be taken with caution, as they do not unequivocally establish a direct correlation between parietal lobe activity and P_T_ or accuracy. In this context, it is conceivable that the progressive intensification of negatively EVES not only augments readiness for processing subsequent discourses (i.e., increased P_T_), but may also foster a different mode of processing.

Concluding, this study suggests that individuals need to engage their cognitive resources to maintain stability and resist stress when faced with EVES. The impact of the EVES can vary greatly depending on the person’s evaluation of a presented stimulus according to the context in which he/she operates (Balconi et al., 2015a,b; Calbi et al., 2022; Mirabella et al., 2023; Montalti and Mirabella, 2023), aligning with the appraisal theories of emotions (Moors and Fischer, 2019; Scherer and Moors, 2019).

However, these results warrant further examination. Future research with larger samples and counterbalancing the sequence of events are necessary to fully explore participants’ reactions. Contrary to our expectations, the accuracy of the aT did not change with increasing negative EVES (Supplementary Figure 1), indicating that the cognitive performance was not affected by the emotional valence of the discourses. Thus, all interpretations tying emotional valence to cognitive performance were based on the correlations of the aT performance with Reg_Stress_ and Res_Stress_. This might indicate that the task was not sufficiently challenging to reveal the impact of emotional valence on cognitive performance or that participants progressively familiarized with the experimental set up. Furthermore, it’s unclear if EEG and behavioral differences during the Pp_1–5_ are due to initial speech requests or progressive intensification of negative valence. However, it appears plausible that the observed differences in emotional response are primarily due to the stress condition (EVES) rather than task difficulty changes from REQs, as the EVES’s negative valence intensification was specifically designed to increment perceived stress levels.

Notwithstanding these limitations, this approach can be used to measure the impact of EVES in diverse contexts and with varying demands, including occupational settings, political sector, clinical environments, marketing, and advertising.

## Data availability statement

The raw data supporting the conclusions of this article will be made available by the authors, without undue reservation.

## Ethics statement

The studies involving humans were approved by the Ethics Committee of the Department of Psychology, Catholic University of the Sacred Heart, Milan, Italy. The studies were conducted in accordance with the local legislation and institutional requirements. The participants provided their written informed consent to participate in this study. Written informed consent was obtained from the individual(s) for the publication of any potentially identifiable images or data included in this article.

## Author contributions

MB: Conceptualization, Methodology, Project administration, Resources, Supervision, Validation, Writing – review & editing. KR: Conceptualization, Data curation, Formal analysis, Investigation, Methodology, Software, Visualization, Writing – original draft.
