# Supplementary material for: Does emotional valence affect cognitive performance and neurophysiological response during decision making? A preliminary study
Source: Front Neurosci. 2024 Aug 9;18:1408526. doi: 10.3389/fnins.2024.1408526 (PMC11341406; doi:10.3389/fnins.2024.1408526)
Supplement: Supplementary file 1 [file Data_Sheet_1.ZIP › Supplementary material/Supplementary file.docx]

**Supplementary file 1**

*SST examining committee’s reactions (video).* D_1_: Friendly examining committee; D_2_: Neutral examining committee; D_3_: Bored examining committee; D_4_: Growing manifestation of impatience examining committee; D_5_: Adverse examining committee.

*Reg_Stress_ and Res_Stress_ scores.* The calculation of Reg_Stress_ score, is coherent for stress regulation due to its consideration of temporal dynamics. This approach captures fluctuations in stress management abilities over multiple time periods. On the other hand, the Res_Stress_ score is designed to assess the subject’s need to adapt their work pace in response to varying stress levels. This is determined by analyzing the differences between D_4_ and D_5_, where D_5_ reflects the subject’s response to increased stress. This Res_Stress_ index signifies the necessity for a change in pace, indicating the impact of stress in all instances.

*Perception of Stress During the Job Interview.* To confirm that participants perceived the job interview situation as stressful, post-experiment questions were administered. Results indicated that 24 out of 26 participants (92%) reported feeling stressed during the test, while 2 participants did not feel excessively stressed. Participants identified various indicators of stress, such as hand sweating, a sensation of heat, accelerated heartbeat, stressful timing, confusion or mental blankness, and changes in voice (e.g., tremor).

*Standardization of Video Stimuli.* Each video used in the study was between 14 to 17 seconds long, featuring a panel of two individuals (one male, one female). Videos were presented in color. To ensure technical consistency, five videos were processed using DaVinci Resolve and Adobe Premiere Pro to standardize resolution (1920x1080), frame rate, bitrate, and format (H.264 to MP4, 16:9 aspect ratio). Audio levels were normalized, and audio compression was standardized (AAC) using Audacity.

*Validation of Video Content and REQ.* An external academic committee comprising three experts in the psychology of emotions and stress evaluated the videos for content and construct validity. This evaluation included semantic analysis and control for confounding variables. Observational behavioral measures were also employed, involving the recording and analysis of representative sample behavior while watching the videos. These measures were compared with content analysis results to ensure robustness.

Furthermore, to ensure the robustness of the REQs in eliciting emotional salience, observational behavior measures were collected during the administration of the REQs. These measures included emotional level, engagement, and perceived Stress, which were coded by trained raters. The results of the coding showed that the observed behaviors were consistent with the expected emotional responses for each REQ.

*Arithmetic Tasks.* The similarity of arithmetic tasks was assessed by two external judges in a pre-experiment phase. A representative sample (N=131) participated in this phase to ensure task equivalence.

As with the representative sample (N=131), no significant differences were found between aT_1-5_ during the five different discourses for the current sample (N=26). The graphical display is presented below:





Figure 1 - The bar graph shows the accuracy index scores for each aT. Bars represent ± 1 Standard Error. No significant differences were found between aT1-5 during the five different discourses

*Data Suitability and Exclusion.* Preliminary analysis considered the statistics of suitable speeches compared to the total. Within the representative sample (N=26), a quantitative and qualitative analysis identified only 1 speech out of 130 as non-conforming (Subject ID “S013” for REQ2), resulting in a 0.769% data exclusion rate.

*Marker, data accuracy and EEG epoch selection.* For each specific phase of the experiment, two markers (indicating the start and end of each phase) were manually inserted by the researcher. Only data from the preparation phase were considered in subsequent analyses. The accuracy of the markers was verified using behavioral results saved by the administration platform. The selection of EEG epochs for analysis was conducted with careful consideration of individual subject preparation times and the removal of segments containing artifacts (e.g. visual, muscular, or other artifacts).

*Power Spectral densities (PSD)*


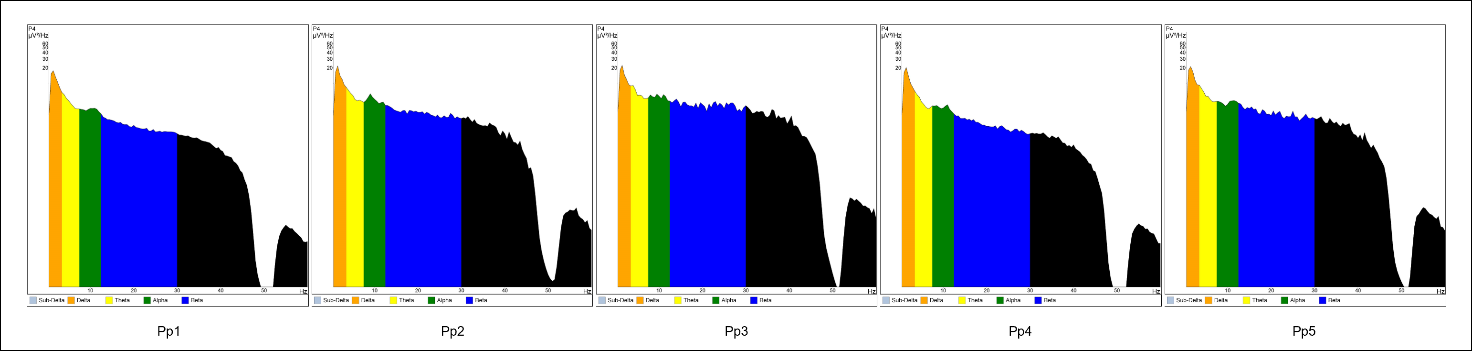


Figure 2 - Power Spectral Densities. Plot of power spectral densities (PSD) across frequency at P4 for each of the five discourses.

*Gender Effect Analysis.* A preliminary sample (N=131) was analyzed to exclude the gender effect, yielding a significance level higher than 0.05. The analysis was replicated in the current study sample (N=26), showing no significant gender differences. Thus, the gender effect was excluded from subsequent analyses to maintain the statistical power of the ANOVA.

*Autonomic data processing and results.* Data concerning cardiovascular (heart rate - HR, heart rate variability - HRV, inter-beat interval - IBI, blood volume pulse - BVP, and PVA) and electrodermal activity (skin conductance level - SCL, and skin conductance response - SCR) were recorded using a wireless Biofeedback_2000_Xpert system with a MULTI radio module (Schuhfried GmbH, Modling, Austria). The multipurpose integrated sensor was placed in correspondence to the distal phalanx of the second finger of the non-dominant hand. Data were sampled at a frequency of 40 Hz. After qualitative and quantitative inspection of data to detect and remove recording or biological artifacts, autonomic activity collected during the baseline and the Pp_1-5_ of the SST was segmented and averaged to calculate mean condition-specific SCL, SCR, BVP, PVA and HR modulations. Inter-beat interval (IBI) metrics were computed starting from raw HR data, meanwhile HRV was computed as the standard deviation of IBI, with the aim of acquiring information that corresponds to the vagal influence on cardiovascular activity (Mendes, 2009).

For autonomic data, six repeated measures ANOVAs with Preparation (5) as within-subject independent variable were applied to the following autonomic indices considered as dependent variables: SCL, SCR, BVP, PVA, HR, and HRV.

For PVA, a significant main effect was revealed for Preparation *(F*_(2.6, 64.7)_ = 6.530, *p* = .001, *η^2^* = .207), with higher mean values (decreasing of negative absolute values) for the Pp_2_ (*p* = .011), Pp_4_ (*p* = .033), and Pp_5_ (p = .043) compared to the Pp_1_. All the descriptive statistics are reported in the supplementary Table 1.





Figure 3 - Autonomic results. The bar graph shows significant differences for PVA for each Pp. Bars represent ± 1 Standard Error and stars (*) mark statistically significant comparisons.

No other significant results were found for SCL, SCR, BVP, HR, HRV, and IBI.

An increase in PVA - involved in the fight or flight response and correlated with latent stress situations - is observed with the emotional salience of stimuli, especially in Pp_5_. The distinctiveness of this response might suggest the possible engagement of more automated processes and a more pronounced involvement of peripheral, rather than central, mechanisms with increasing REQs.
