# Supplementary material for: Does emotional valence affect cognitive performance and neurophysiological response during decision making? A preliminary study
Source: Front Neurosci. 2024 Aug 9;18:1408526. doi: 10.3389/fnins.2024.1408526 (PMC11341406; doi:10.3389/fnins.2024.1408526)
Supplement: Supplementary file 1 [file Data_Sheet_1.ZIP › Supplementary material/Supplementary Table1.docx]

**Table S1.** **(a)** Descriptive statistics for behavioural data: the stress regulation index (Reg_Stress_); the stress resilience index (Res_Stress_); the raw data of the preparation times (P_T_) for the five discourses (Pp_1-5_); the accuracy index for the five arithmetic task (aT_1-5_). **(b)** Descriptive statistics for EEG Pp_1-5_ data. **(c)** Descriptive statistics for autonomic Pp_1-5_ data.

**Table s1(a)**

| Variable | Mean | Standard deviation |
| --- | --- | --- |
| Reg_Stress_ | 7.062 | 1.592 |
| Res_Stress_ | 6.462 | 2.611 |
| P_Traw1_ | 55.267 | 26.188 |
| P_Traw2_ | 34.358 | 22.341 |
| P_Traw3_ | 38.764 | 24.041 |
| P_Traw4_ | 44.118 | 32.986 |
| P_Traw5_ | 29.848 | 25.907 |
| accuracy index aT_1_ | 0.755 | 0.326 |
| accuracy index aT_2_ | 0.769 | 0.274 |
| accuracy index aT_3_ | 0.935 | 0.112 |
| accuracy index aT_4_ | 0.790 | 0.242 |
| accuracy index aT_5_ | 0.743 | 0.293 |

**Table s1(b)**

| Electrode - Preparation Phase (Pp) | **Delta** | | **Theta** | | **Alpha** | | **Beta** | | **Gamma** | |
| --- | --- | --- | --- | --- | --- | --- | --- | --- | --- | --- |
|  | Mean | Standard deviation | Mean | Standard deviation | Mean | Standard deviation | Mean | Standard deviation | Mean | Standard deviation |
| AFz_Pp1 | 1.278 | 2.048 | 1.211 | 1.708 | 0.512 | 1.419 | 0.927 | 2.379 | 2.454 | 9.076 |
| Fp1_Pp1 | 1.558 | 2.645 | 1.673 | 3.381 | 0.146 | 0.796 | 0.477 | 1.684 | 0.789 | 2.581 |
| F7_Pp1 | 1.008 | 1.339 | 0.856 | 1.109 | -0.026 | 0.337 | 0.361 | 1.245 | 0.540 | 1.849 |
| F3_Pp1 | 0.733 | 1.094 | 0.499 | 0.592 | -0.102 | 0.355 | 0.142 | 0.592 | 0.327 | 1.061 |
| Fz_Pp1 | 0.637 | 1.194 | 0.289 | 0.628 | -0.178 | 0.327 | 0.120 | 0.679 | 0.648 | 1.863 |
| F4_Pp1 | 0.687 | 1.259 | 0.361 | 0.581 | -0.073 | 0.368 | 0.177 | 0.512 | 0.417 | 0.796 |
| F8_Pp1 | 0.752 | 1.373 | 0.501 | 0.942 | -0.004 | 0.376 | 0.249 | 0.659 | 0.484 | 1.400 |
| Fp2_Pp1 | 1.089 | 1.919 | 0.818 | 1.460 | 0.018 | 0.676 | 0.195 | 0.923 | 0.366 | 1.749 |
| T7_Pp1 | 0.859 | 1.470 | 0.400 | 0.534 | -0.063 | 0.315 | 1.285 | 2.953 | 2.693 | 5.218 |
| C3_Pp1 | 0.945 | 2.681 | 0.235 | 0.578 | -0.232 | 0.394 | 0.067 | 0.573 | 0.678 | 1.804 |
| Cz_Pp1 | 0.676 | 2.158 | 0.113 | 0.480 | -0.227 | 0.285 | 0.055 | 0.534 | 0.528 | 1.613 |
| C4_Pp1 | 0.727 | 2.233 | 0.172 | 0.680 | -0.221 | 0.354 | 0.055 | 0.563 | 0.614 | 1.819 |
| T8_Pp1 | 0.505 | 0.843 | 0.233 | 0.439 | -0.011 | 0.282 | 1.322 | 2.490 | 2.588 | 5.571 |
| P3_Pp1 | 0.904 | 3.471 | 0.173 | 0.615 | -0.189 | 0.450 | 0.426 | 1.323 | 1.162 | 2.337 |
| Pz_Pp1 | 0.605 | 2.374 | 0.132 | 0.530 | -0.246 | 0.374 | 0.105 | 0.541 | 0.770 | 1.845 |
| **P4_Pp1** | **-0.315** | **0.490** | **-0.234** | **0.504** | **0.138** | **1.260** | **1.644** | **2.066** | **5.012** | **5.503** |
| O1_Pp1 | 2.387 | 2.983 | 1.654 | 0.895 | 0.663 | 1.132 | 0.553 | 1.087 | 1.160 | 2.470 |
| O2_Pp1 | 0.393 | 0.646 | 0.639 | 0.635 | 0.594 | 0.927 | 1.481 | 1.576 | 2.598 | 3.106 |
| AFz_Pp2 | 1.612 | 2.776 | 1.528 | 1.874 | 0.557 | 1.083 | 0.843 | 1.419 | 1.736 | 3.098 |
| Fp1_Pp2 | 1.414 | 2.676 | 1.153 | 1.650 | 0.150 | 0.789 | 0.372 | 1.037 | 0.960 | 2.183 |
| F7_Pp2 | 1.547 | 3.136 | 1.016 | 1.297 | 0.077 | 0.431 | 1.024 | 2.548 | 2.315 | 6.658 |
| F3_Pp2 | 0.823 | 1.294 | 0.571 | 0.866 | -0.016 | 0.561 | 0.305 | 0.889 | 1.299 | 2.957 |
| Fz_Pp2 | 0.510 | 1.089 | 0.288 | 0.652 | -0.135 | 0.503 | 0.124 | 0.586 | 0.534 | 1.115 |
| F4_Pp2 | 0.748 | 1.221 | 0.495 | 0.695 | 0.035 | 0.508 | 0.398 | 0.619 | 1.287 | 1.884 |
| F8_Pp2 | 0.935 | 1.208 | 0.616 | 0.794 | 0.164 | 0.578 | 0.741 | 1.417 | 1.579 | 3.298 |
| Fp2_Pp2 | 1.210 | 2.161 | 1.018 | 1.485 | 0.077 | 0.682 | 0.360 | 1.213 | 0.759 | 2.135 |
| T7_Pp2 | 0.895 | 1.550 | 0.368 | 0.697 | 0.011 | 0.498 | 2.100 | 4.598 | 3.402 | 7.378 |
| C3_Pp2 | 0.425 | 0.802 | 0.278 | 0.697 | -0.043 | 1.140 | 0.173 | 0.610 | 0.855 | 1.509 |
| Cz_Pp2 | 0.272 | 0.648 | 0.172 | 0.541 | -0.033 | 0.687 | 0.164 | 0.543 | 0.538 | 0.901 |
| C4_Pp2 | 0.318 | 0.772 | 0.269 | 0.732 | -0.003 | 0.836 | 0.301 | 1.008 | 0.781 | 1.523 |
| T8_Pp2 | 0.935 | 1.231 | 0.427 | 0.555 | 0.248 | 0.734 | 1.186 | 1.313 | 2.357 | 3.378 |
| P3_Pp2 | 0.214 | 0.628 | 0.189 | 0.618 | 0.043 | 0.805 | 0.540 | 1.369 | 1.028 | 1.293 |
| Pz_Pp2 | 0.129 | 0.506 | 0.143 | 0.555 | -0.023 | 0.669 | 0.190 | 0.585 | 0.711 | 0.920 |
| **P4_Pp2** | **0.258** | **0.675** | **0.288** | **0.807** | **0.125** | **0.932** | **0.302** | **0.711** | **0.878** | **1.063** |
| O1_Pp2 | 1.037 | 2.034 | 0.738 | 0.994 | 1.077 | 1.407 | 1.664 | 1.469 | 2.528 | 2.637 |
| O2_Pp2 | 0.833 | 1.081 | 0.804 | 0.796 | 1.543 | 2.249 | 2.231 | 1.843 | 3.391 | 3.828 |
| AFz_Pp3 | 0.656 | 1.065 | 0.730 | 1.111 | 0.247 | 0.893 | 0.837 | 1.552 | 1.043 | 1.856 |
| Fp1_Pp3 | 0.605 | 0.985 | 0.587 | 0.948 | 0.001 | 0.575 | 0.549 | 1.265 | 0.915 | 1.729 |
| F7_Pp3 | 0.633 | 1.156 | 0.439 | 0.574 | -0.035 | 0.325 | 0.495 | 0.892 | 0.699 | 1.480 |
| F3_Pp3 | 0.422 | 0.509 | 0.265 | 0.331 | -0.089 | 0.307 | 0.456 | 1.159 | 1.212 | 3.070 |
| Fz_Pp3 | 0.294 | 0.782 | 0.226 | 0.725 | -0.176 | 0.389 | 0.251 | 0.820 | 0.602 | 1.222 |
| F4_Pp3 | 0.379 | 0.713 | 0.288 | 0.448 | 0.005 | 0.424 | 0.404 | 0.641 | 0.905 | 1.152 |
| F8_Pp3 | 0.376 | 0.749 | 0.331 | 0.520 | 0.048 | 0.376 | 0.654 | 0.964 | 0.896 | 1.420 |
| Fp2_Pp3 | 0.565 | 1.006 | 0.576 | 1.000 | 0.019 | 0.591 | 0.591 | 1.524 | 0.969 | 2.113 |
| T7_Pp3 | 0.739 | 1.003 | 0.337 | 0.606 | 0.067 | 0.537 | 2.061 | 3.984 | 2.852 | 5.251 |
| C3_Pp3 | 0.292 | 0.668 | 0.247 | 0.721 | -0.188 | 0.548 | 0.350 | 1.024 | 0.961 | 1.610 |
| Cz_Pp3 | 0.156 | 0.559 | 0.126 | 0.527 | -0.189 | 0.362 | 0.194 | 0.698 | 0.566 | 1.013 |
| C4_Pp3 | 0.203 | 0.812 | 0.263 | 1.010 | -0.179 | 0.432 | 0.362 | 1.156 | 0.901 | 1.863 |
| T8_Pp3 | 0.516 | 1.100 | 0.261 | 0.398 | 0.119 | 0.454 | 1.326 | 1.458 | 2.414 | 3.177 |
| P3_Pp3 | 0.116 | 0.468 | 0.159 | 0.641 | -0.040 | 0.813 | 0.747 | 2.055 | 1.302 | 1.878 |
| Pz_Pp3 | 0.052 | 0.451 | 0.099 | 0.517 | -0.224 | 0.392 | 0.173 | 0.633 | 0.703 | 1.041 |
| **P4_Pp3** | **0.110** | **0.612** | **0.178** | **0.669** | **-0.181** | **0.399** | **0.319** | **0.868** | **0.881** | **1.271** |
| O1_Pp3 | 0.328 | 0.513 | 0.492 | 0.629 | 1.102 | 1.437 | 1.750 | 1.638 | 3.020 | 3.976 |
| O2_Pp3 | 0.353 | 0.528 | 0.608 | 0.578 | 1.146 | 1.558 | 1.779 | 1.857 | 2.713 | 3.074 |
| AFz_Pp4 | 0.853 | 1.849 | 1.244 | 2.675 | 0.645 | 1.483 | 2.019 | 5.816 | 2.406 | 4.952 |
| Fp1_Pp4 | 1.130 | 2.714 | 1.266 | 2.060 | 1.370 | 4.844 | 3.466 | 11.271 | 2.109 | 4.545 |
| F7_Pp4 | 1.528 | 3.673 | 1.136 | 1.750 | 0.199 | 0.559 | 0.743 | 1.280 | 1.469 | 2.792 |
| F3_Pp4 | 0.911 | 1.781 | 0.550 | 0.808 | 0.108 | 0.504 | 0.477 | 0.734 | 1.545 | 2.391 |
| Fz_Pp4 | 0.537 | 1.210 | 0.296 | 0.695 | -0.045 | 0.464 | 0.399 | 0.820 | 1.102 | 1.491 |
| F4_Pp4 | 0.537 | 1.199 | 0.401 | 0.676 | 0.118 | 0.545 | 0.582 | 0.831 | 1.408 | 1.434 |
| F8_Pp4 | 1.191 | 2.383 | 0.565 | 0.854 | 0.193 | 0.498 | 0.961 | 1.307 | 1.474 | 1.708 |
| Fp2_Pp4 | 0.957 | 1.889 | 0.979 | 1.811 | 0.192 | 0.790 | 1.025 | 2.404 | 0.960 | 1.782 |
| T7_Pp4 | 1.305 | 2.704 | 0.562 | 0.909 | 0.154 | 0.532 | 2.178 | 3.802 | 3.057 | 4.783 |
| C3_Pp4 | 0.605 | 1.191 | 0.331 | 0.644 | -0.045 | 0.547 | 0.560 | 1.033 | 1.749 | 2.842 |
| Cz_Pp4 | 0.401 | 0.876 | 0.182 | 0.511 | 0.005 | 0.438 | 0.360 | 0.684 | 1.092 | 1.424 |
| C4_Pp4 | 0.446 | 1.056 | 0.245 | 0.542 | -0.023 | 0.435 | 0.732 | 1.649 | 1.602 | 2.233 |
| T8_Pp4 | 1.065 | 2.060 | 0.510 | 0.715 | 0.173 | 0.402 | 1.480 | 2.492 | 2.810 | 4.574 |
| P3_Pp4 | 0.446 | 0.882 | 0.325 | 0.629 | 0.383 | 1.666 | 1.048 | 2.086 | 2.291 | 2.925 |
| Pz_Pp4 | 0.339 | 0.775 | 0.214 | 0.493 | 0.006 | 0.479 | 0.384 | 0.714 | 1.305 | 1.580 |
| **P4_Pp4** | **0.423** | **0.853** | **0.335** | **0.635** | **0.054** | **0.489** | **0.620** | **1.136** | **1.732** | **2.354** |
| O1_Pp4 | 0.893 | 1.361 | 0.952 | 1.567 | 1.126 | 1.281 | 2.562 | 3.610 | 4.886 | 9.329 |
| O2_Pp4 | 0.853 | 1.152 | 1.019 | 1.097 | 1.374 | 1.842 | 2.153 | 2.333 | 3.593 | 5.105 |
| AFz_Pp5 | 1.102 | 2.207 | 1.033 | 1.384 | 0.420 | 0.912 | 0.740 | 1.730 | 1.176 | 2.208 |
| Fp1_Pp5 | 1.138 | 2.050 | 1.084 | 1.411 | 0.249 | 0.854 | 1.286 | 3.595 | 2.026 | 5.081 |
| F7_Pp5 | 1.155 | 1.914 | 1.208 | 1.470 | 0.203 | 0.688 | 0.433 | 0.874 | 0.498 | 1.059 |
| F3_Pp5 | 0.789 | 0.992 | 0.705 | 0.965 | -0.003 | 0.361 | 0.261 | 0.500 | 0.480 | 1.184 |
| Fz_Pp5 | 0.555 | 0.966 | 0.447 | 0.823 | -0.053 | 0.658 | 0.732 | 2.417 | 1.359 | 4.327 |
| F4_Pp5 | 0.581 | 1.026 | 0.513 | 0.577 | 0.011 | 0.402 | 0.465 | 0.968 | 0.855 | 1.575 |
| F8_Pp5 | 0.590 | 1.245 | 0.527 | 0.794 | 0.100 | 0.406 | 0.539 | 0.711 | 0.836 | 1.441 |
| Fp2_Pp5 | 1.106 | 1.994 | 1.037 | 1.425 | 0.296 | 0.965 | 1.347 | 3.485 | 1.815 | 4.693 |
| T7_Pp5 | 0.995 | 1.892 | 0.705 | 1.555 | 0.097 | 0.612 | 1.250 | 2.157 | 1.623 | 3.003 |
| C3_Pp5 | 0.614 | 0.947 | 0.623 | 1.414 | 0.126 | 1.542 | 0.974 | 3.665 | 1.805 | 6.224 |
| Cz_Pp5 | 0.389 | 0.703 | 0.338 | 0.826 | 0.125 | 1.179 | 0.852 | 2.988 | 1.655 | 5.734 |
| C4_Pp5 | 0.478 | 0.898 | 0.551 | 1.377 | 0.285 | 1.985 | 1.504 | 5.142 | 2.643 | 9.335 |
| T8_Pp5 | 0.572 | 0.847 | 0.346 | 0.489 | 0.122 | 0.396 | 0.960 | 1.097 | 1.655 | 2.920 |
| P3_Pp5 | 0.498 | 0.902 | 0.846 | 2.236 | 0.859 | 3.191 | 1.930 | 5.876 | 3.225 | 9.807 |
| Pz_Pp5 | 0.327 | 0.683 | 0.442 | 1.286 | 0.280 | 1.830 | 1.123 | 4.172 | 2.720 | 9.776 |
| **P4_Pp5** | **0.417** | **0.832** | **0.668** | **1.823** | **0.447** | **2.345** | **1.567** | **5.641** | **3.403** | **12.284** |
| O1_Pp5 | 0.413 | 0.803 | 0.507 | 0.581 | 0.958 | 1.357 | 1.737 | 2.683 | 3.338 | 7.300 |
| O2_Pp5 | 0.451 | 0.601 | 0.716 | 0.880 | 1.071 | 1.896 | 1.847 | 2.816 | 2.972 | 6.118 |

**Table s1(c)**

| Prepration Phase | **SCL** | | **SCR** | | **BVP** | | **PVA** | | **HR** | | **HRV** | |
| --- | --- | --- | --- | --- | --- | --- | --- | --- | --- | --- | --- | --- |
|  | Mean | Standard deviation | Mean | Standard deviation | Mean | Standard deviation | Mean | Standard deviation | Mean | Standard deviation | Mean | Standard deviation |
| Pp1 | 2.000 | 2.511 | 6.426 | 10.513 | 0.001 | 0.002 | -0.492 | 0.339 | 0.224 | 0.254 | 0.845 | 1.906 |
| Pp2 | 2.105 | 2.878 | 8.910 | 15.275 | 0.001 | 0.003 | -0.338 | 0.475 | 0.163 | 0.324 | 0.532 | 0.953 |
| Pp3 | 2.247 | 2.864 | 9.340 | 15.174 | 0.001 | 0.003 | -0.353 | 0.446 | 0.197 | 0.344 | 0.549 | 1.324 |
| Pp4 | 2.244 | 3.124 | 8.437 | 15.370 | 0.000 | 0.005 | -0.338 | 0.480 | 0.162 | 0.298 | 0.557 | 1.221 |
| Pp5 | 2.516 | 3.684 | 8.978 | 15.370 | 0.001 | 0.004 | -0.331 | 0.448 | 0.105 | 0.334 | 0.387 | 0.921 |
