# Supplementary material for: Does emotional valence affect cognitive performance and neurophysiological response during decision making? A preliminary study
Source: Front Neurosci. 2024 Aug 9;18:1408526. doi: 10.3389/fnins.2024.1408526 (PMC11341406; doi:10.3389/fnins.2024.1408526)
Supplement: Supplementary file 1 [file Data_Sheet_1.ZIP › Supplementary material/Supplementary Table2.docx]

**Table S2.** Analysis of variance (ANOVA) table for P4 (Delta, Theta, Alpha, Beta, and Gamma band) in the preparation phase (Pp) for the five discourses (Pp_1-5_)

| Electrode × Preparation | | | | | | | |
| --- | --- | --- | --- | --- | --- | --- | --- |
| Electrodes | (I) Preparation Phase | (J) Preparation Phase | p-value (Delta) | p-value (Theta) | p-value (Alpha) | p-value (Beta) | p-value (Gamma) |
| P4 | Pp1 | Pp2 | **0.001** | **0.003** | 1.000 | 0.062 | **0.025** |
|  |  | Pp3 | **0.012** | **0.017** | 1.000 | 0.084 | **0.031** |
|  |  | Pp4 | **0.003** | **0.001** | 1.000 | 0.424 | 0.156 |
|  |  | Pp5 | **0.006** | 0.269 | 1.000 | 1.000 | 1.000 |
|  | Pp2 | Pp1 | **0.001** | **0.003** | 1.000 | 0.062 | **0.025** |
|  |  | Pp3 | 1.000 | 1.000 | 1.000 | 1.000 | 1.000 |
|  |  | Pp4 | 1.000 | 1.000 | 1.000 | 1.000 | 0.706 |
|  |  | Pp5 | 1.000 | 1.000 | 1.000 | 1.000 | 1.000 |
|  | Pp3 | Pp1 | **0.012** | **0.017** | 1.000 | 0.084 | **0.031** |
|  |  | Pp2 | 1.000 | 1.000 | 1.000 | 1.000 | 1.000 |
|  |  | Pp4 | 0.896 | 1.000 | **0.044** | 1.000 | 1.000 |
|  |  | Pp5 | 0.318 | 1.000 | 1.000 | 1.000 | 1.000 |
|  | Pp4 | Pp1 | **0.003** | **0.001** | 1.000 | 0.424 | 0.156 |
|  |  | Pp2 | 1.000 | 1.000 | 1.000 | 1.000 | 0.706 |
|  |  | Pp3 | 0.896 | 1.000 | **0.044** | 1.000 | 1.000 |
|  |  | Pp5 | 1.000 | 1.000 | 1.000 | 1.000 | 1.000 |
|  | Pp5 | Pp1 | **0.006** | 0.269 | 1.000 | 1.000 | 1.000 |
|  |  | Pp2 | 1.000 | 1.000 | 1.000 | 1.000 | 1.000 |
|  |  | Pp3 | 0.318 | 1.000 | 1.000 | 1.000 | 1.000 |
|  |  | Pp4 | 1.000 | 1.000 | 1.000 | 1.000 | 1.000 |
