# Supplementary figures and images for: Does emotional valence affect cognitive performance and neurophysiological response during decision making? A preliminary study
Source: Front Neurosci. 2024 Aug 9;18:1408526. doi: 10.3389/fnins.2024.1408526 (PMC11341406; doi:10.3389/fnins.2024.1408526)

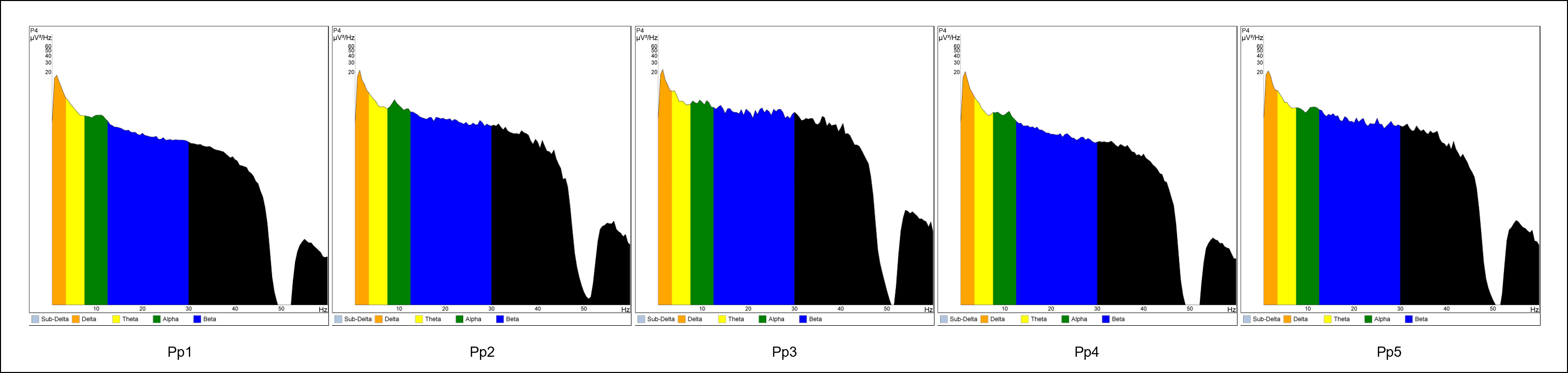

Supplement: Supplementary file 1 [file Data_Sheet_1.ZIP › Supplementary material/Supplementary Figure/FigureSUP2.tiff]
